# Two Cases of Mediastinal Non-Seminomatous Germ Cell Tumor with Complete Resection by Robot-Assisted Thoracoscopic Surgery after Induction Chemotherapy: Techniques for Identification and Preservation of the Phrenic Nerve

**DOI:** 10.70352/scrj.cr.25-0383

**Published:** 2025-09-09

**Authors:** Ryuji Nakamura, Katsuhiro Okuda, Kensuke Iguchi, Shin Hosokawa, Emi Hagui, Kensuke Chiba, Tsutomu Tatematsu, Keisuke Yokota

**Affiliations:** Department of Thoracic and Pediatric Surgery, Graduate School of Medical Sciences and Medical School, Nagoya City University, Nagoya, Aichi, Japan

**Keywords:** mediastinal non-seminomatous germ cell tumor, robot-assisted thoracoscopic surgery, phrenic nerve, induction chemotherapy, indocyanine green

## Abstract

**INTRODUCTION:**

Mediastinal non-seminomatous germ cell tumors (NSGCTs) are rare tumors. Neoadjuvant chemotherapy followed by complete surgical resection of residual masses is recommended, and is often performed through a median sternotomy or thoracotomy with regard to the influence of induction chemotherapy and tumor size. We herein report 2 cases of mediastinal NSGCT that were surgically resected by robot-assisted thoracoscopic surgery (RATS) using the subxiphoid approach.

**CASE PRESENTATION:**

Case 1: A 23-year-old man was diagnosed with an anterior mediastinal mass measuring 95 × 73 × 73 mm while undergoing an examination due to fever. He was diagnosed with a yolk sac tumor based on percutaneous needle biopsy. After 4 cycles of neoadjuvant chemotherapy, the patient underwent tumor resection combined with wedge resection of the left upper lung via a robot-assisted subxiphoid approach. It was particularly difficult to identify the left phrenic nerve because of stiff adhesions and thickening of the tissue. Therefore, we decided to perform dissection of tissue other than that surrounding the left phrenic nerve. Subsequently, the thymus and tumor were flipped into the left thoracic cavity, and the left phrenic nerve was easily identified and preserved from the pericardial side. The patient was discharged without any postoperative complications. Case 2: An 18-year-old man was diagnosed with a yolk sac tumor measuring 86 × 68 × 150 mm during an examination to investigate intermittent right chest pain. After 4 cycles of neoadjuvant chemotherapy, the patient underwent anterior mediastinal tumor resection via a robot-assisted subxiphoid approach. The tumor was close to the right pulmonary hilum, and the inflammation was so intense that it was difficult to identify the right phrenic nerve. In this case, indocyanine green fluorescence imaging was helpful for identifying the right phrenic nerve. The tumor was completely resected. The patient was discharged on POD 6 without any postoperative complications.

**CONCLUSIONS:**

We report 2 cases of mediastinal NSGCT after induction chemotherapy that were completely resected using RATS. The use of techniques to accurately identify the phrenic nerve and the advantages of robot-assisted surgery via the subxiphoid approach enabled safe and minimally invasive surgical procedures.

## Abbreviations

AFP
: alpha-fetoprotein
BEP
: bleomycin, etoposide, and cisplatin
GCT
: germ cell tumor
ICG
: indocyanine green
ICS
: intercostal space
IGCCCG
: International Germ Cell Cancer Collaborative Group
NSGCT
: non-seminomatous germ cell tumor
OS
: overall survival
RATS
: robot-assisted thoracoscopic surgery

## INTRODUCTION

Mediastinal GCTs represent nearly 1%–4% of mediastinal tumors.^1,2)^ Prognostic factors have been defined by the IGCCCG consensus classification.^3)^ According to the IGCCCG classification, mediastinal NSGCTs have a poor prognosis, and the 5-year OS rate has been reported to be 27.3%–51%.^4–9)^ Typically, 4 cycles of conventional-dose cisplatin-based chemotherapy followed by complete surgical resection of residual masses are recommended. Mediastinal NSGCTs are often resected by thoracotomy because of the large tumor size, influence of induction chemotherapy, and invasion of surrounding organs. Complete resection has been reported in up to 86% of patients with mediastinal NSGCT.^10)^ RATS has several advantages, including 3D visualization and the use of articulated joint forceps with more degrees of freedom of motion.^11)^ Moreover, the subxiphoid approach has several advantages, providing a wide visualization of the entire mediastinum and both thoracic cavities while enabling a bilateral procedure.^12)^ Here, we describe 2 cases of mediastinal NSGCT after induction chemotherapy followed by complete resection by RATS.

## CASE PRESENTATION

### Case 1

**Fig. 1:**
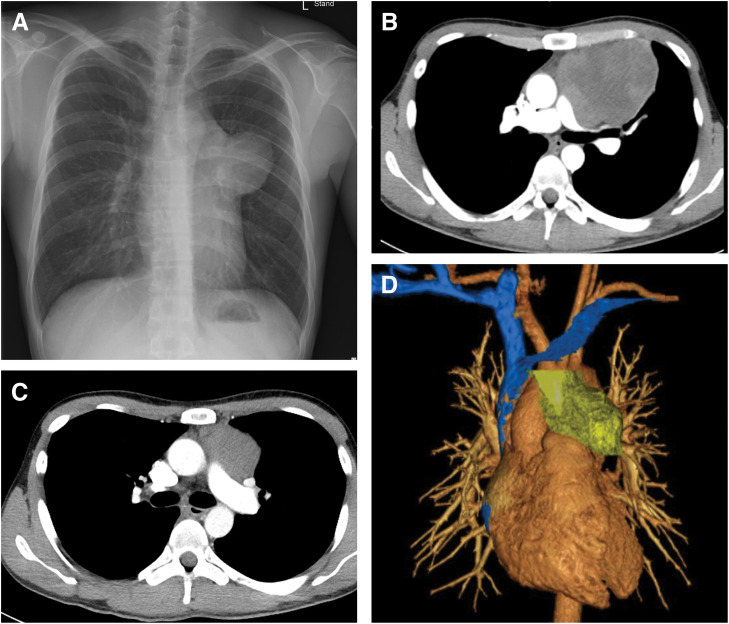
(**A**) Initial chest X-ray showing an opacity projecting into the left thoracic cavity. (**B**) Chest CT before chemotherapy showing a large mass in the anterior mediastinum. (**C**) Chest CT after chemotherapy showing that the mass had shrunk. (**D**) 3D reconstruction image before surgery.

**Fig. 2:**
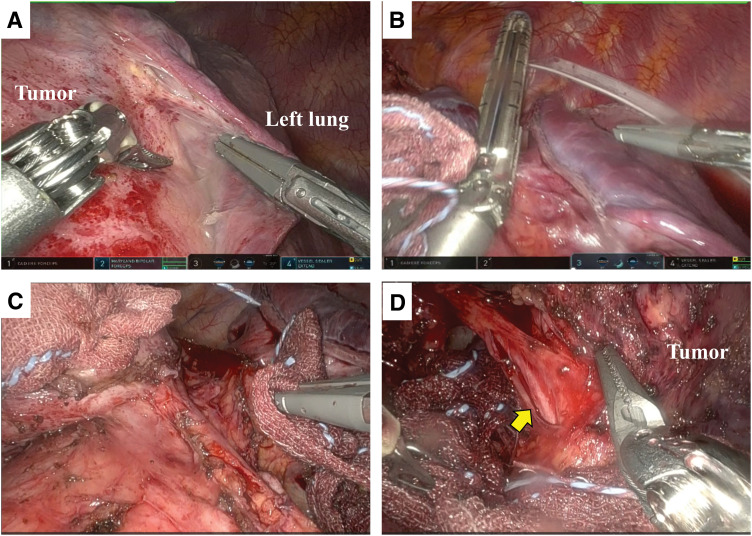
Intraoperative findings. (**A**) The tumor adhered firmly to the anterior segment of the left upper lobe. (**B**) Wedge resection of the left upper lung with a stapler. (**C**) Tight adhesions behind the tumor and poor vision due to the presence of the tumor prevented dissection of the phrenic nerve from the side of the thoracic cavity. (**D**) The thymus and tumor were flipped into the left thoracic cavity. Dissection was performed from the pericardial side to preserve the left phrenic nerve (arrow).

The patient was a 23-year-old man with no relevant medical history. The patient was diagnosed with an anterior mediastinal mass while undergoing an examination due to fever. Chest radiography during the initial examination revealed an abnormal shadow projecting into the left thoracic cavity (**Fig. 1A**). Chest CT showed a large mass in the anterior mediastinum measuring 95 × 73 × 73 mm and extensive compression of the heart and left lung (**Fig. 2B**). Blood analysis showed an elevated AFP level of 396 ng/mL. We performed echo-guided percutaneous needle biopsy as soon as possible, and a yolk sac tumor was diagnosed. He was started on chemotherapy with BEP (cisplatin [20 mg/m^2^] on days 1–5, etoposide [100 mg/m^2^], bleomycin [30 mg/bodyweight] on days 2, 9, and 16; every 3 weeks). After the 1st cycle, the patient developed febrile neutropenia, and etoposide was continued with a 25% dose reduction from the 2nd cycle. At the end of 4 cycles, the tumor had shrunk to 43 × 29 × 36 mm, and the AFP level had decreased to within the normal range (**Fig. 1C** and **1D**). An operation was planned on day 40 of the 4th cycle. After general anesthesia, the patient was intubated with a double-lumen tube for one-lung ventilation. The patient was placed in the supine position. We performed the operation via a robot-assisted subxiphoid approach using 4 incisions, including a 3-cm longitudinal main incision under the subxiphoid and 2 ports on the midclavicular line and the anterior axillary line at the left 6th ICS, and 1 port on the midclavicular line at the right 6th ICS. The 30-degree robotic camera was inserted into the left 6th ICS port on the midclavicular line (the 3rd arm) to secure the view around the left pulmonary hilum. The da Vinci Xi System (Intuitive Surgical, Sunnyvale, CA, USA) was used. The procedure was performed using 4 robotic arms and a CO_2_ insufflation system under 8 mmHg of pressure. The tumor adhered firmly to the anterior segment of the left upper lobe, raising suspicion of tumor invasion (**Fig. 2A**). Therefore, we performed wedge resection of the left lung using a stapler (**Fig. 2B**). The left phrenic nerve was identified on the caudal side of the tumor. However, it was particularly difficult to identify the left phrenic nerve due to the stiff adhesions and thickened tissue around the tumor (**Fig. 2C**). The pericardium was firmly adherent, but could be dissected from the tumor. Subsequently, the thymus and tumor were flipped into the left thoracic cavity and the left phrenic nerve was identified and preserved from the pericardial side (**Fig. 2D**). This process minimizes the risk of damage and ensures preservation. The tumor was completely resected. The operation time was 300 min with 28 mL of blood loss.

A histopathological examination revealed the absence of viable cells. The patient was discharged from the hospital on the POD 5 without any postoperative complications, and was alive 1 year and 6 months after the surgery without recurrence.

### Case 2

**Fig. 3:**
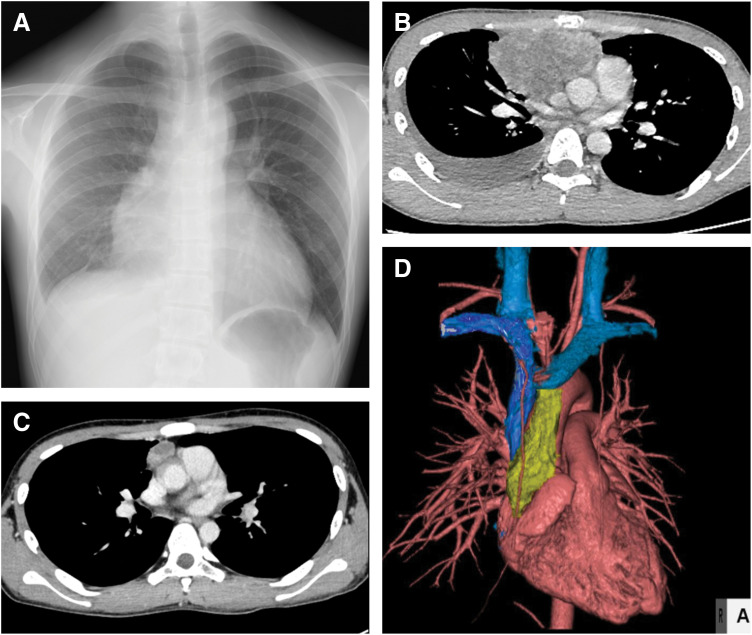
(**A**) Chest X-ray was taken on admission showed widening of the lower mediastinum. (**B**) Chest CT before chemotherapy showing a large mass in the anterior mediastinum and right pleural effusion. (**C**) Chest CT after chemotherapy showing that the mass had shrunk. (**D**) 3D reconstruction image before surgery.

**Fig. 4:**
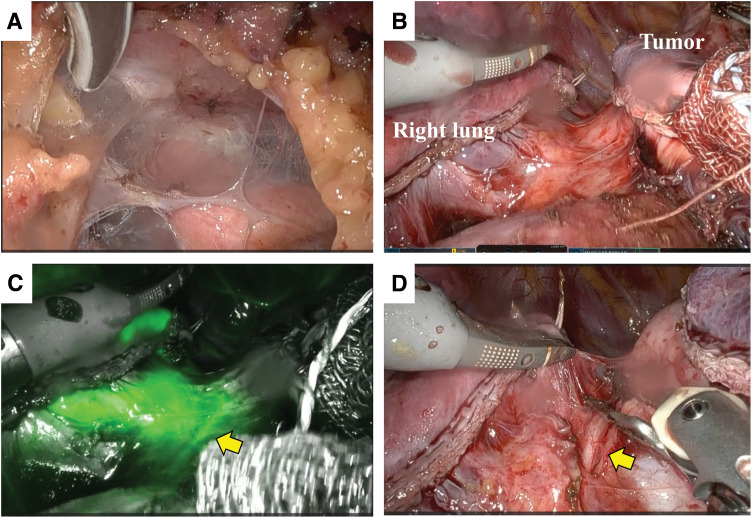
Intraoperative findings. (**A**) The right thoracic cavity was completely adherent. (**B**) The tumor was close to the right pulmonary hilum and intense inflammation was present. It was difficult to identify the right phrenic nerve. (**C**) The right phrenic nerve was identified using ICG fluorescence (arrow). (**D**) The right phrenic nerve was identified at the site visualized by ICG fluorescence (arrow). ICG, indocyanine green

The patient was an 18-year-old male. The patient presented with intermittent right chest pain. Chest radiography revealed widening of the lower mediastinum (**Fig. 3A**). Chest CT showed an anterior mediastinal tumor measuring 86 × 68 × 150 mm and right pleural effusion (**Fig. 3B**). Thoracic drainage was performed and pleural fluid cytology was negative. A laboratory test revealed an AFP level of 30565.9 ng/mL. Therefore, he was clinically diagnosed with a yolk sac tumor, and chemotherapy was initiated with BEP (cisplatin [20 mg/m^2^] on days 1–5, etoposide [100 mg/m^2^], bleomycin [30 mg/bodyweight] on days 2, 9, and 16; every 3 weeks). He completed 4 cycles without severe adverse events. This regimen was effective against his tumor, which was 18 × 14 × 66 mm before the operation, with AFP level decreasing to within the normal range (**Fig. 3C** and **3D**). An operation was planned on day 34 of the 4th cycle. We performed the operation via a robot-assisted subxiphoid approach in 4 incisions, including a 3-cm longitudinal main incision under the subxiphoid and 2 ports on the midclavicular line and the anterior axillary line at the left 6th ICS, and 1 port on the midclavicular line at the right 6th ICS. The patient was placed in the supine position, and CO_2_ was insufflated at 8 mmHg. The tumor mainly extended to the right side, so the camera port was placed in the subxiphoid. The right thoracic cavity was completely adherent (**Fig. 4A**). The tumor was widely adherent to the right lung, but wedge resection of the right upper and middle lobes was possible. The tumor was close to the right pulmonary hilum, and the inflammation was so intense that it was difficult to identify the right phrenic nerve (**Fig. 4B**). Therefore, we decided to use ICG fluorescence imaging. A total of 5 mL of ICG solution, prepared by dissolving one 25 mg vial of ICG (Diagnogreen; Daiichi Sankyo, Tokyo, Japan) in 10 mL saline, was intravenously injected. This allowed identification of the right phrenic nerve, which was visualized using ICG fluorescence (**Fig. 4C** and **4D**). The tumor and thymus were subsequently dissected from the innominate vein and pericardium, which was firmly adherent to the tumor. The tumor was completely resected. The operation time was 285 min, with 4 mL of blood loss. The patient was discharged on POD 6 without any postoperative complications.

A histopathological examination revealed a yolk sac tumor that was completely resected and 10% residual tumor cells. Based on the results of multidisciplinary conferences and after obtaining informed consent from the patient and his family, the decision was made not to administer consolidation chemotherapy. The patient is alive 9 months after surgery without recurrence.

## DISCUSSION

Mediastinal NSGSCs are associated with a poor prognosis, with reported 5-year OS rates of 27.3%–51%.^4–9)^ Regarding prognostic factors, Sakurai et al. reported that elevated preoperative serum tumor marker levels and the presence of viable cells in the resected specimens were significantly associated with recurrence in a study of 48 patients with resected primary mediastinal malignant GCTs.^4)^ Sarkaria et al. also reported on 57 patients undergoing primary resection of mediastinal NSGCT. Their multivariate analysis identified normalizing or decreasing preoperative tumor markers as independent predictors of better OS, and limited pathological stage and the absence of cancer cells in the residual tumor also approached statistical significance.^5)^ In our cases, the preoperative tumor marker levels had normalized, but one patient had 10% viable cells in the resected specimen. According to the European consensus conference on diagnosis and treatment of germ cell cancer: a report of the second meeting of the European Germ Cell Cancer Consensus Group: part II, if a completely resected tumor presents >10% viable cancer, or if completeness of the resection is in doubt, consolidation chemotherapy might be justified.^13)^ Therefore, we decided to follow-up the patient without additional chemotherapy.

The number of patients with mediastinal primary NSGCT is limited, and many of them are operated on by median sternotomy or thoracotomy because of the influence of induction chemotherapy and tumor size. Therefore, there are few reports on robot-assisted surgery.^14,15)^ At our institution, the indications for robot-assisted resection of anterior mediastinal tumors are as follows: (1) a good surgical field can be maintained, meaning that this procedure is not indicated for tumors exceeding 10 cm in short diameter located in the midline of the mediastinum or lodged in the cranial side of the left brachiocephalic vein; (2) anatomical lung resection is not required; and (3) large vessel reconstruction is not required. We believe that robot-assisted surgery allowed us to perform safe and minimally invasive surgery because of the good view of the pulmonary hilum and capture of the fine layered structures. These advantages made the operation more delicate (e.g., dissection from the pericardium and phrenic nerve) in our cases.

The clinical use of ICG for visualization of the thoracic duct, phrenic nerve, sentinel lymph nodes, pleural nodules, and intersegmental planes has been reported.^16,17)^ It was difficult to identify the phrenic nerve when inflammation in the pulmonary hilum was intense. Additionally, the risk of phrenic nerve injury increases if the tissue is firm. In these cases, fluorescence imaging with ICG was useful for identification and preservation of the phrenic nerve. Additionally, by flipping the tumor into the thoracic cavity and approaching it from the pericardial side, where the tissue is relatively soft, the phrenic nerve can be safely dissected and preserved.

## CONCLUSIONS

We report 2 cases of mediastinal NSGCT that were completely resected by RATS after induction chemotherapy. The use of techniques to accurately identify the phrenic nerve and the advantages of robot-assisted surgery via the subxiphoid approach enabled safe and minimally invasive surgical procedures.

## DECLARATIONS

### Availability of data and materials

Data sharing is not applicable to this article, because datasets were neither generated nor analyzed for case reports.
